# Loop-mediated isothermal amplification-lateral flow dipstick (LAMP-LFD) for detection of wine microorganisms

**DOI:** 10.1007/s11274-026-05066-x

**Published:** 2026-06-05

**Authors:** Abdul Ghani, Sergi Ferrer

**Affiliations:** ENOLAB, BioTecMed, Edifici Investigació 3.71. Universitat de València c/ Dr. Moliner 50, Burjassot-València, 46100 Spain

**Keywords:** Lateral-flow dipstick, LAMP, Wine fermentation, Panbacteria, Panfungal, Rapid detection

## Abstract

Wine is undoubtedly one of the oldest and most celebrated beverages across cultures. It is primarily crafted from grapes through a fermentation process where yeast transforms sugars into alcohol. The presence of unwanted microorganisms can cause spoilage, so early detection of microorganisms is crucial. The previously reported conventional methods and molecular methods have so many drawbacks they are time consuming and expensive to be used in wine industries. Therefore, this study presents a novel broad-range loop-mediated isothermal amplification coupled with lateral flow dipstick (LAMP-LFD) assay for the rapid detection of wine-related microorganisms. The novelty of this work lies in the design and modification broad-range LAMP primer sets, labelled with biotin and fluorescein isothiocyanate, enabling simultaneous detection of wine spoilage microorganisms, including acetic acid bacteria and *Brettanomyces* fungi, as well as beneficial wine fermentation microorganisms such as lactic acid bacteria and *Saccharomyces cerevisiae*, with immediate visualisation of amplification products on the LFD strip. The results illustrate that the designed primers were specific for wine-related microorganisms at optimised conditions in the LAMP-LFD. The newly developed method for detecting wine-related microorganisms was very sensitive, and it can detect 10^2^ CFU/mL, which is more sensitive than commercial polymerase chain reaction (PCR). This developed LAMP-LFD detection method is an essential advancement for the wine industry, which offers a reliable and efficient means to protect wine quality. We believe that the method is a promising proof-of-concept tool that could be further developed and validated for use in the wine industry.

**Supplementary Information:**

The online version contains supplementary material available at 10.1007/s11274-026-05066-x.

## Introduction

Wine is a complex product produced through a biotechnological process called fermentation. This process converts sugars into alcohol by yeast (James et al. 2023). There are two primary fermentation processes involved in wine production: alcoholic and malolactic fermentation (Maicas 2021). Although yeast plays the main role in wine fermentation, some other fungi and lactic acid bacteria also contribute to the distinctive qualities of wine (Alba-Lois and Segal-Kischinevzky 2010). Malolactic fermentation, carried out by lactic acid bacteria, converts malic acid into lactic acid and CO₂, reducing acidity and contributing to sensory changes and microbiological stability when properly controlled (Nguyen and Nguyen 2024). Furthermore, microbial metabolism can also modify grape-derived phenolic compounds, thereby influencing wine colour, aroma, mouthfeel, and overall quality (Lopez-Velez at al. 2003).

In addition, certain bacteria and fungi generate secondary metabolites that influence wine quality (Huang et al. 2023). The most common wine spoilage microorganisms include lactic acid bacteria (genera *Lactobacillus*,* Pediococcus*, and *Leuconostoc*), acetic acid bacteria (genera *Acetobacter* and *Gluconobacter*), and yeast (genera *Brettanomyces* and *Candida*) (Bartowsky et al. 2003, 2009; Woolfit et al. 2007). During malolactic fermentation, lactic acid bacteria generate metabolites that alter flavour and aroma (Peyer et al. 2015). Although acetic acid bacteria are not used in fermentation, they can produce compounds like acetaldehyde and ethyl acetate, which are significant in wine spoilage (Bartowsky et al. 2009). Some filamentous fungi can also spoil wine by producing mycotoxins, such as aflatoxins, fumonisins, and ochratoxins, which harm wine consumers (Welke 2019).

A key focus in wine production is ensuring safety and protecting human health. The main parameters for assessing wine spoilage include colour change, turbidity, and undesirable odours (Rêgo et al. 2022). Some conventional methods for monitoring wine microorganisms are now considered as outdated. For e.g. culturing and microscopy are time-consuming. Molecular methods, such as polymerase chain reaction (PCR), are among the most commonly used techniques for microorganism detection in the wine industry (Kačániová et al. 2012). Despite its advantages, PCR has notable drawbacks, such as being time-consuming and expensive. Globally, new methods for microorganisms’ detection are being developed, providing faster and more portable solutions. One of these method is loop-mediated isothermal amplification (LAMP), which is faster and more affordable than PCR (Deconinck et al. 2023). LAMP uses four to six primers, although primer designing is tricky and it needs careful consideration, but the primary advantage of LAMP lies in its amplification protocol, which operates at a constant temperature and produces rapid results without the need for costly thermal cycler, which makes it simple and affordable (Wang et al. 2022). Additionally, LAMP products can be visualised using various methods, including turbidity, colourimetric dye detection and gel electrophoresis. However, colourimetric methods have some drawbacks, as the colour change is not obvious at low amplification Pang et al. (2019), and gel electrophoresis can be a lengthy process.

So far, many studies reported of LAMP reaction for detection of wine related microorganisms (Soares-Santos et al. 2018; Ghani and Ferrer 2025), but designing broad range LAMP primers and use of integrated LAMP with the lateral flow dipstick (LAMP-LFD) is still lacking. Based on this, we aim to develop the LAMP-LFD method for the rapid detection of wine related microorganisms. In this study we designed and modified four different LAMP primer sets: Panbacteria (Wine related bacteria), Panfungal (Wine related fungi), one specific for *Oenococcus* spp. and one for *Saccharomyces cerevisiae*, were selected in this study because of their essential roles in wine fermentation and quality control. *Oenococcus* spp. are primarily responsible for malolactic fermentation, a crucial process that improves wine stability and sensory characteristics by converting malic acid into lactic acid. Similarly, *S. cerevisiae* is the principal yeast involved in alcoholic fermentation, converting sugars into ethanol and contributing significantly to wine aroma and flavor development. We designed these LAMP primers using primer designing tools such as Primer Explorer V5 and GLAPD/ and labelled these primers with biotin and fluorescein isothiocyanate (FITC) for LAMP-LFD.

We also used gold nanoparticles (AuNPs) to reduce the chances of false positive amplification in LAMP amplification (Jang and Kim 2022; Ghani and Ferrer 2025). We assessed the specificity, detection limit, and reproducibility of the LAMP-LFD method, comparing its efficacy against other molecular methods, such as PCR, from the literature. This study demonstrate the effectiveness of the LAMP-LFD approach in ensuring wine safety and quality within the wine industry.

## Materials and methods

### Strains and wine samples

Important wine related bacterial strains such as *Gluconobacter oxydans* 4629, *Oenococcus oeni* 4042, *Acetobacter aceti* CECT 298^T^, *Lactiplantibacillus plantarum* 5458 and several fungal strains, including *Saccharomyces cerevisiae* CECT 2056, *Hanseniaspora uvarum* 5380, *Metschnikowia pulcherrima* 5163, *Lachancea thermotolerans* 5387 and *Brettanomyces bruxellensis* 1560 were obtained from different culture collections used in this study. All the strains were cultivated in red wine (Bobal) and then extracted DNA and performed LAMP-LFD.

### DNA extraction

This study employed the “broken-cell method,” a technique used to break the cell walls of microorganisms to extract DNA. The cells inoculated into the wine were centrifuged at 13,000 rpm for 3 min and washed twice with milli-U water and then washed with a 10% TEN solution (0.1 M Tris HCl, pH 7.5; 0.05 M EDTA; and 0.8 M NaCl) with 2% PVP (Wt. 360,00 g/mol) (Sigma). Finally, the cells were washed twice with milli-U water and then dissolved in TE buffer (0.1 M Tris HCl, pH 7.5, and 0.05 M EDTA), after that they were vortexed for ten minutes at maximum limit (Soares-Santos et al. 2018).

### Primer designing and validation

In this study, we used four different groups of primers: two sets for bacteria and two for fungi. One of the bacterial primer sets is general bacterial primer, which can amplify (*Lactiplantibacillus* spp., *Gluconobacter* ssp., *Acetobacter* spp., and *Oenococcus* spp., while the other is specific to *Oenococcus* genus designed in this study. Similarly, we have one general primer set designed by Inácio et al. (2008), for yeast and another specific for *Saccharomyces cerevisiae* designed in this study. The general primers are intended to detect wine bacteria and yeast, while the specific primers target only *Oenococcus* and *Saccharomyces* species. The bacterial primers were designed based on the 16S rRNA gene, and the fungal primers were based on the ITS region, using Primer Explorer V5 and GLAPD software. Although these primers set are similar like those used in LAMP, with some modifications: biotin is labelled on the inner forward primers, and FITC is labelled on the inner backward primers. The LAMP-LFD primers were validated using Primer-BLAST and MEGA-X alignment.

### LAMP reaction conditions and optimization

DNA was extracted from all the strains used in this research as mentioned in DNA extraction method. A total reaction system volume of 25 µL was prepared, which included the following components: 1.4 mM of dNTPs (NZYtech), 0.2 µM of each outer primer (Metabion), 1.6 µM of each inner primer, 0.4 µM of each loop primer, 8 mM of MgSO_4_, 1× Isothermal Amplification Buffer, 0.32 U/µL of *Bst* polymerase 2.0 (New England BioLabs), 7.5 µL of extracted DNA, and sterile Milli-U water used for the negative control. To optimise of the LAMP reaction for each primer, we performed LAMP reaction at 62 °C for 30 min with 5 µL of 5 nm diameter gold nanoparticles (Sigma Aldrich), from our previous study (Ghani and Ferrer 2025).

### LAMP-lateral flow dipstick (LAMP-LFD) assay validation

**Fig. 1:**
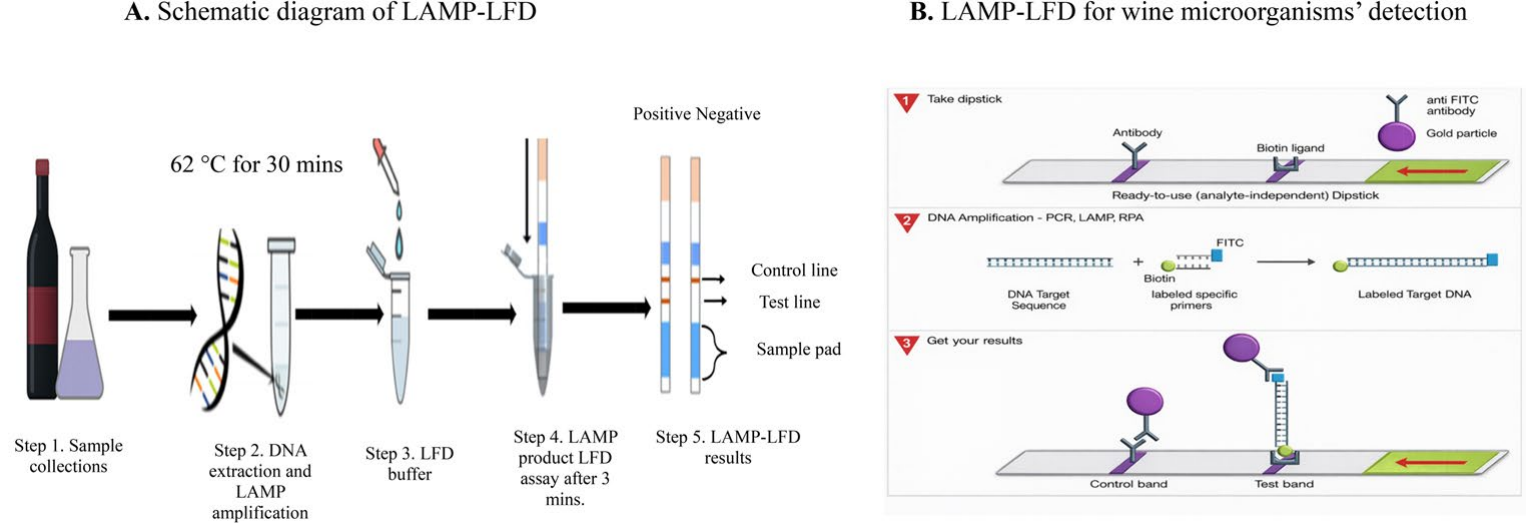
The flowchart (**A**) schematic diagram of LAMP-LFD (**B**) components and principle of LFD

For the LAMP-LFD reaction, the BIP primer was replaced with a 5′-biotinylated BIP primer and FIP with FITC at the optimized concentratins. The reaction was incubated at 62 °C for 30 min. To detect the biotinylated LAMP products using lateral flow dipsticks (LFD), a commercial rapid dipstick produced by Milenia Biotec GmbH (Milenia^®^ GenLine HybriDetect, Germany) was utilised. These dipsticks capture the biotinylated LAMP products and detect them by gold nanoparticles that bind the FITC tag of the amplified DNA in the test line. The control line at the top of the stick features immobilised antibodies as shown in Fig. 1. After completing the LAMP reaction, 5 µL of the LAMP products were transferred to the LFD strip and placed in 80 µL of running buffer provided by the test kit (Milenia biotec). Finally, the LFD strip was immersed in the buffer provided by the producer for 3 min, and the detection results were evaluated by observing the test and control lines on the LFD strips.

### LAMP-LFD specificity and repeatability

DNA was extracted from all the wine spoilage microorganisms mentioned above, and the LAMP reaction was carried out under optimised conditions as mentioned in LAMP reaction. The amplified products were evaluated using lateral flow devices (LFD) and subjected to 2% gel electrophoresis. The reactions were repeated three times to confirm the repeatability of results obtained from the LAMP-LFD assay.

### LAMP-LFD detection limit

For determination of detection limit of the LAMP-LFD method we used various initial viable cell serial dilution 10^8^, 10^6^, 10^4^, and 10^2^ CFU/mL were tested in replicate reactions using wine related microorganisms in LAMP reaction. Finally resulting amplification products were analysed using LFD strips and 2% agarose gel electrophoresis.

## Results

### Primer designing and validation

We designed and modified four sets of primers targeting different groups of wine microorganisms: Panbacteria (wine related bacteria), Panfungal (wine related fungi), *Oenococcus* (genus-specific), and *Saccharomyces cerevisiae* (species-specific). The primers were designed based on the 16S rRNA gene for bacteria and the ITS region for fungi. We utilised GLAPD and Primer Explorer V5 primer designing software for designing primers in this study, as illustrated in Table 1. The *in-silico* designed primers were validated using Primer-BLAST and MEGA-X for sequence alignment.

**Table 1:** Comprehensive overview of all developed LAMP-LFD primers, detail target species, utilized softwares, and specific target genes

Primers	Target Microorganisms	References	Target gene	Software
Panbacteria-F3 AGGTGGGGATGACGTCAAG	1. *O. oeni* 4042 2. *L. plantarum* 5458 3. *G. oxydans* 4629 4. *A. aceti* CECT 298^T^	1. Enolab collection 2. Enolab collection 3. Enolab collection 4. CECT collection	16S rRNA	GLAPD
Panbacteria-B3 CGGGAACGTATTCACCGC				
Panbacteria-FIP CTAGCTTCCCACTGTCACCGCTCCTCATGGCCCTTATGTCC				
Panbacteria-BIP AACCGTCTCAGTTCGGATTGCATCCGCGATTACTAGCGATTC				
Panbacteria-LF AGCACGTGTGTAGCCCA				
Panbacteria-LB CTCTGCAACTCGAGTGCATG				
Panfungal-F3 GCATATCAATAAGCGGAGGAAAAG	1. *S. cerevisiae* CECT 2056 2. *B. bruxellensis* 1560 3. *M. pulchernima* 5163 4. *L. thermotolerans* 5387 5. *H. uvarum* 5380	1.CECT collection 2. Enolab collection 3. Enolab collection 4. Enolab collection 5. Enolab collection	ITS region	Primer Explorer V5
Panfungal-B3 CCTTCCCTTTCAACAATTTCAC				
Panfungal-FIP CTGCATTCCCAAACAACTCGACTCACAGAGGGTGAGAATCCCG				
Panfungal-BIP TATTGGCGAGAGACCGATAGCGTTTCACTCTCTTTTCAAAGTTC				
L2OoF3 GTGGGGGATAACATTTGGAA	*Oenococcus oeni* 4042	Enolab collection	16S rRNA	GLAPD
L2OoB3 CGTAGGAGTTTGGGCAGT				
L2OoFIP3 GCAAGACCATCCTCTAGCGATCACCGCGTAACAACAAATCAC				
L2OoBIP3 TAGGGTAGAAGCCTACCAAGGCTCTCAGTCCCAATGTGGC				
L2OoLF AAGGACCTTTCAAAGAGATCAC				
L2OoLB AATGATGCGTAGCCGAGTTG				
SCM1LF2-F3 TGACCAAGTACGATTACTGGCC	*Saccharomyces cerevisiae* CECT 2056	CECT collection	ITS region	Primer Explorer V5
SCM1LF2-B3 GTTGTATGGTACATTGTTTGCATCT				
SCM1LF2-FIP GGCAGAACCTTGTGATTTTCTTCTACCAAAGTGGAACCTGCACATCG				
SCM1LF2-BIP ACTGTCGATTATTGAAATAGACGAACTTAATGGAATACTGTTTAACCTGCT				
SCM1LF2-LF CACTTATATGTAGCTCTTTGTAGGC				

### LAMP assay specificity

Comparative experiments performed under identical amplification conditions demonstrated that the inclusion of AuNPs reduced non-specific amplification and improved assay specificity, whereas reactions without AuNPs occasionally produced false-positive signals. These comparative results are presented in Supplementary Fig. S1, confirming the beneficial role of AuNPs in enhancing assay reliability.

### LAMP-LFD assay validation

**Fig. 2:**
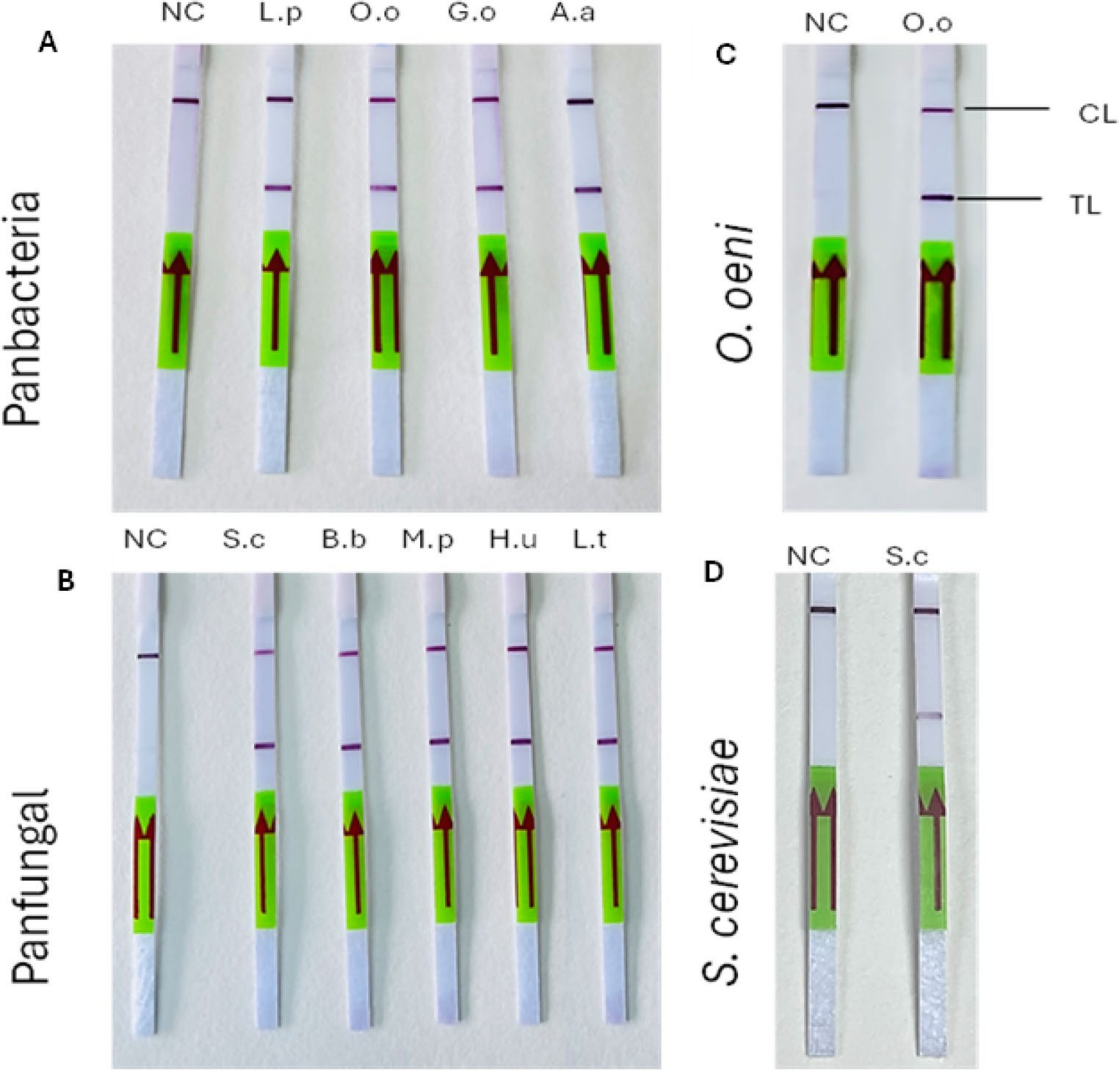
The LAMP-LFD reaction process of all selected wine microbes were conducted using designed primer sets. The results indicate that negative controls (NC) used only showed the control line band (CL), and all other microbial DNA with related primers set showed the control band and test line band (TL). (**A**) Panbacteria primers used in the LFD-LAMP reaction. (**B**) Panfungal primer sets used in LAMP-LFD. (**C**) *Oenococcus* spp., designed primers used in LAMP-LFD and (**D**) *S. cerevisiae* primers used in LFD-LAMP reaction. L.p =. *L. plantarum* 5458, O.o = *O. oeni* 4042, G.o = *G. oxydans* 4629, A.a *= A. aceti* CECT 298^T^, S.c = *S. cerevisiae* CECT 2056, B.b = *B. bruxellensis* 1560, M.p = *M. pulchernima* 5163, H.u = H. *uvarum* 5380, L.t = L. *thermotolerans* 5387

**Fig. 3:**
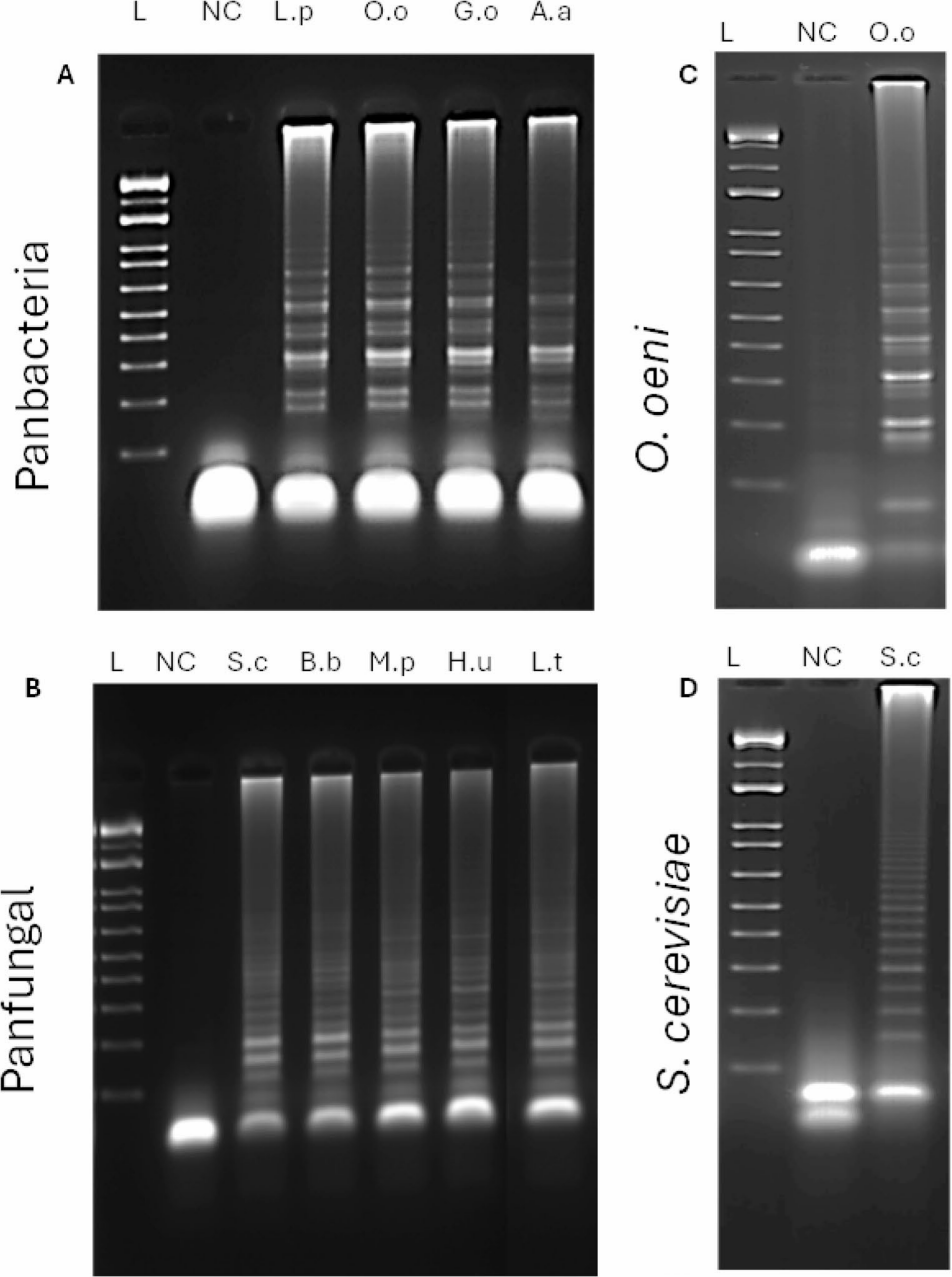
The amplified products from the LAMP-LFD primer were analysed through 2% gel electrophoresis. The gel includes 1 kb plus molecular ladder (L) to assist in determining the sizes of the amplified fragments. The designation NC indicates negative control. (**A**) Wine-related bacteria amplified using Panbacteria primers with LAMP-LFD reaction. (**B**) Wine-related yeast and fungal amplified by LAMP-LFD using Panfungal primers (**C**) *Oenococcus* spp., designed primers in LAMP-LFD, and (**D**) *S. cerevisiae* primers used in LAMP-LFD reaction

DNA was extracted from all the wine microorganisms selected in this study at a 6 × 10^8^ CFU/mL viable cell concentrations and used as templates in the LAMP reaction. The LAMP products, tested with all the designed primer sets, were examined using lateral flow dipsticks. Two bands on the LFD strip indicated positive amplification. In contrast, a single band suggested no amplification, as shown in Fig. 2. For further confirmation of LAMP-LFD, we used gel electrophoresis which displayed a ladder pattern or smear with positive and showed no band in negative control, as illustrated in Fig. 3.

### LAMP-LFD specificity

**Fig. 4:**
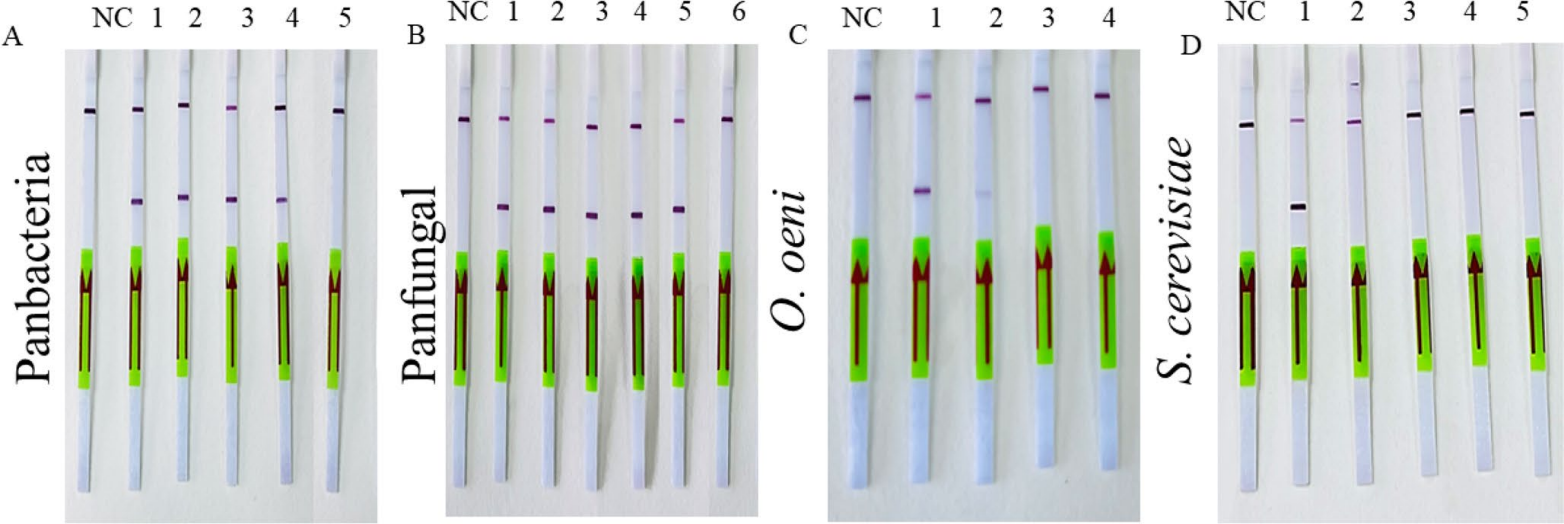
The specificity of LAMP-LFD detection for wine microorganisms. NC= Negative control, (**A**) Panbacteria primers set, 1 = *L. plantarum 5458*, 2 = *O. oeni* 4042, 3 = *G. oxydans* 4629, 4 = *A. aceti* CECT 298^T^, 5 = *S. cerevisiae* CECT 2056. (**B**) Panfungal primers set, 1 = *S. cerevisiae* CECT 2056, 2 = *B. bruxellensis* 1560, 3 = *M. pulchernima* 5163, 4 = *L. thermotolerans* 5387, 5 = *H. uvarum* 5380, 6 = *O. oeni* 4042. (**C**) *Oenococcus* spp., specific primers set, 1 = *O. oeni* 4042, 2 = *L. plantarum* 5458, 3 = *G. oxydans* 4629, 4 = *A. aceti CECT 298*^*T*^. (**D**) *S. cerevisiae* specific LAMP-LFD primers set, 1 = *S. cerevisiae* CECT 2056, 2 = *B. bruxellensis* 1560, 3 = *M. pulchernima* 5163, 4 = *L. thermotolerans* 5387, 5 = *H. uvarum* 5380

The specificity of the designed primers were evaluated using LAMP-LFD with three types of templates: the corresponding target DNA, a non-specific DNA template, and a negative control (no DNA). For all primer sets, a visible test line on the LFD strip appeared only when the reaction contained the specific target DNA. No test lines were observed with non-specific DNA or negative controls shown in Fig. 4. These results confirm that each primer set selectively amplified its intended target without cross-reactivity.

**Fig. 5:**
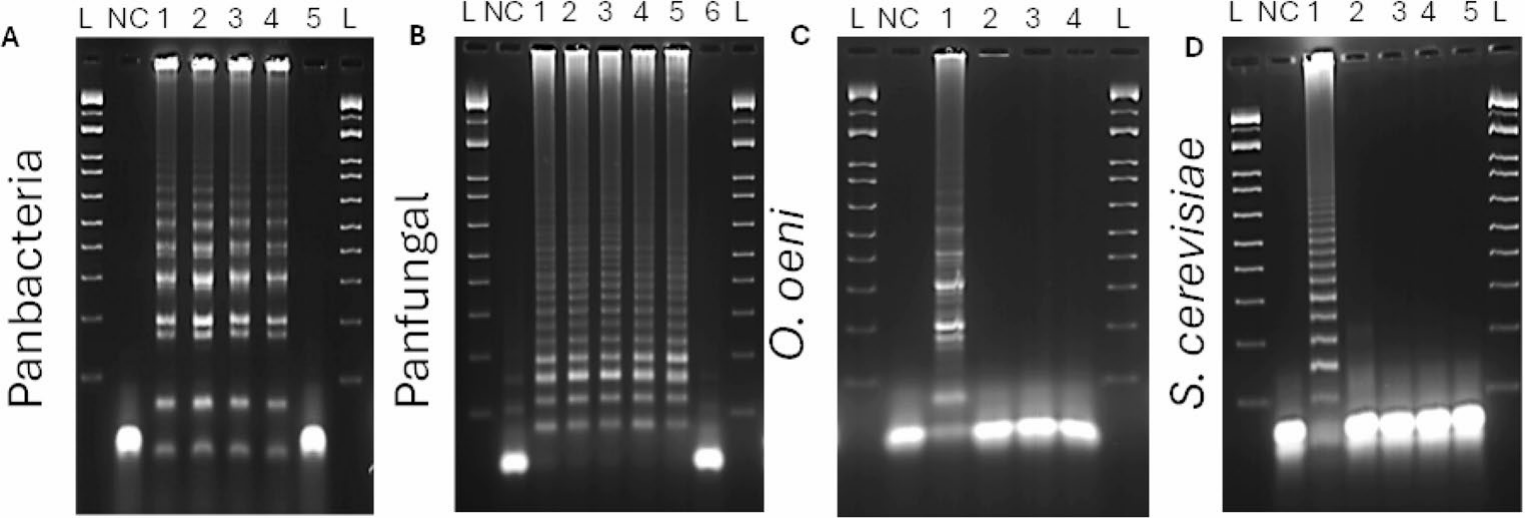
The specificity of the LAMP-LFD assay was confirmed by gel. L= ladder, (**A**) Panbacteria primers set, 1 = *L. plantarum 5458*, 2 = *O. oeni* 4042, 3 = *G. oxydans* 4629, 4 = *A. aceti* CECT 298^T^, 5 = *S. cerevisiae* CECT 2056. (**B**) Panfungal primers set, 1 = *S. cerevisiae* CECT 2056, 2 = *B. bruxellensis* 1560, 3 = *M. pulchernima* 5163, 4 = *L. thermotolerans* 5387, 5 = *H. uvarum* 5380, 6 = *O. oeni* 4042. (**C**) *Oenococcus* spp., specific primers set, 1 = *O. oeni* 4042, 2 = *L. plantarum* 5458, 3 = *G. oxydans* 4629, 4 = *A. aceti CECT 298*^*T*^. (**D**) *S. cerevisiae* specific LAMP-LFD primers set, 1 = *S. cerevisiae* CECT 2056, 2 = *B. bruxellensis* 1560, 3 = *M. pulchernima* 5163, 4 = *L. thermotolerans* 5387, 5 = *H. uvarum* 5380

To further verify our results, the LAMP products were analyzed by 2% agarose gel electrophoresis. Amplification bands were observed exclusively with reactions containing the specific target DNA, while no amplification was detected in negative or non-specific controls as shown in Fig. 5. Together, these results demonstrate the high specificity of the designed primer sets.

### LAMP-LFD detection limit

**Fig. 6:**
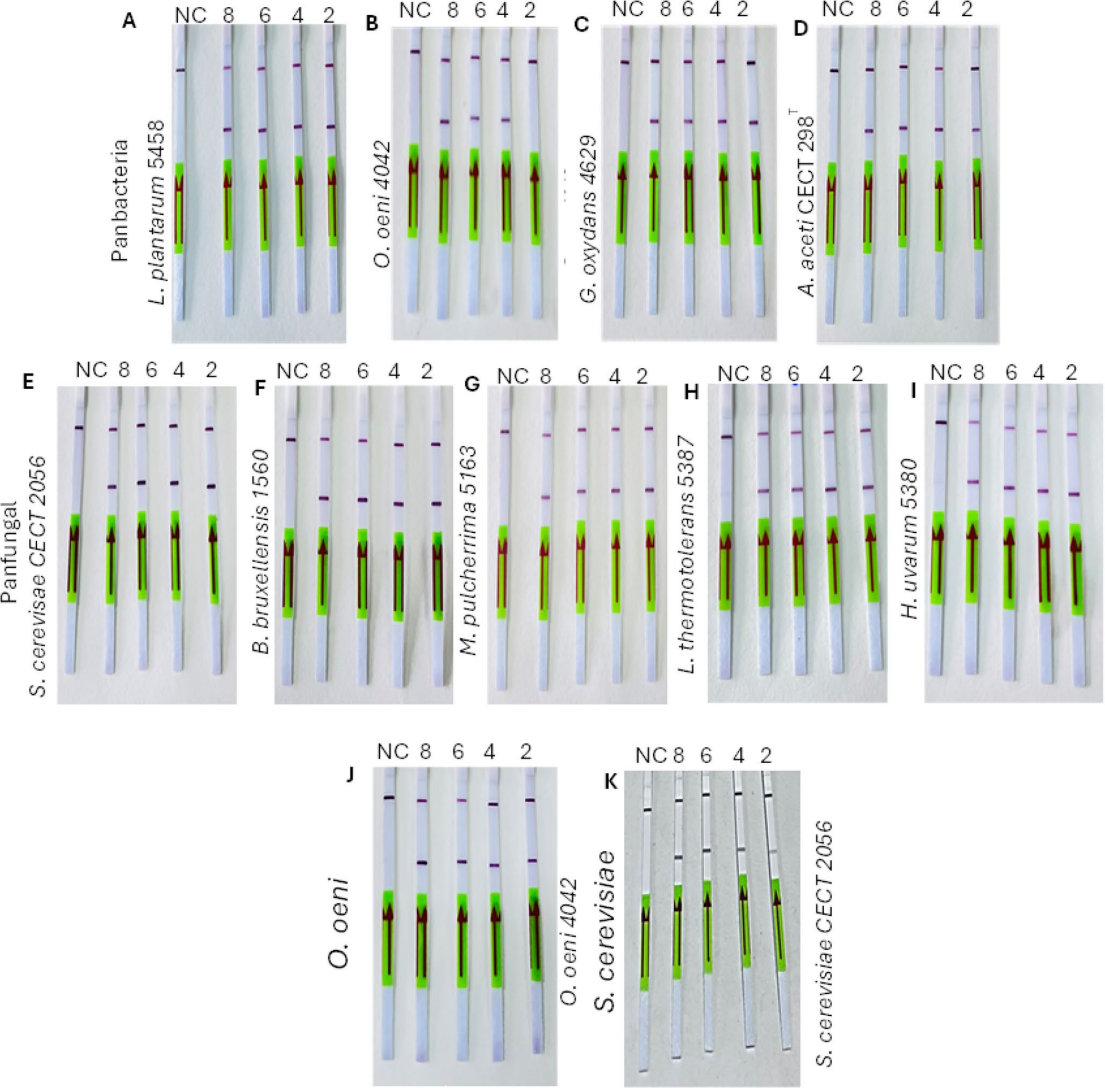
Strips showing the detection limit of LAMP-LFD method with different primers for detection of wine microorganisms. NC = negative control, 8 = 10^8^, 6 = 10^6^, 4 = 10^4^, and 2 = 10^2^ CFU/mL. Panbacteria primers used for bacterial species (**A** = *L. plantarum 5458*, **B** = *O. oeni* 4042, **C** = *G. oxydans* 4629, **D** = *A. aceti* CECT 298^T^). Panfungal primers for yeast (**E** = *S. cerevisiae* CECT 2056, **F** = *B. bruxellensis* 1560, **G** = *M. pulcherrima* 5163, **H** = *L. thermotolerans* 5387, **I** = *H. uvarum* 5380), *Oenococcus* spp., specific primers (**J** = *O. oeni* 4042) and *S. cerevisiae* primers (**K** = *S. cerevisiae* CECT 2056)

**Fig. 7:**
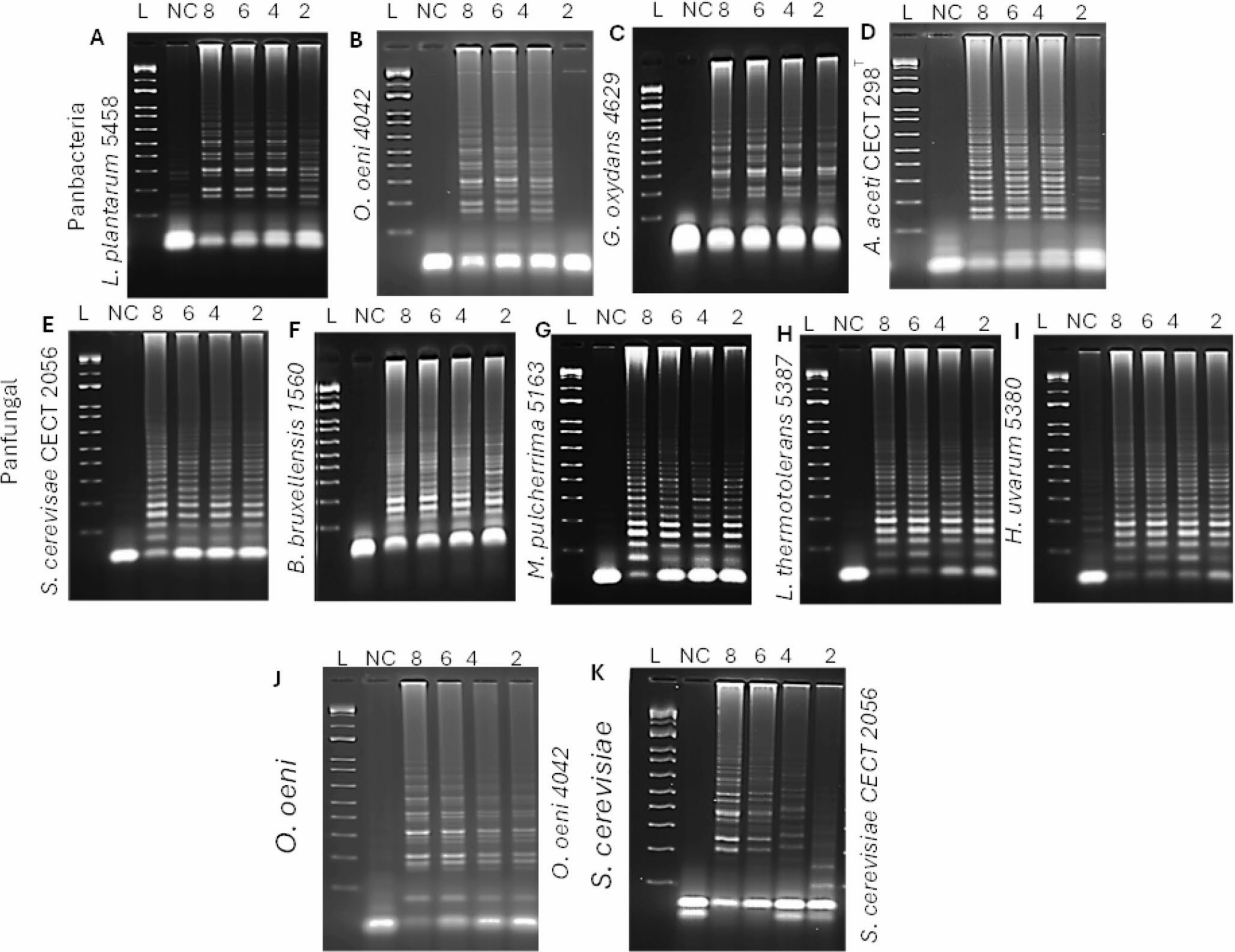
Detection limit of LAMP-LFD for detecting wine microorganisms on 2% agarose gel electrophoresis. L=ladder. NC = negative control, 8 = 10^8^, 6 = 10^6^, 4 = 10^4^, and 2 = 10^2^ CFU/mL. Panbacteria primers used for bacterial species (**A** = *L. plantarum 5458*, **B** = *O. oeni* 4042, **C** = *G. oxydans* 4629, **D** = *A. aceti* CECT 298^T^). Panfungal primers for yeast (**E** = *S. cerevisiae* CECT 2056, **F** = *B. bruxellensis* 1560, **G** = *M. pulcherrima* 5163, **H** = *L. thermotolerans* 5387, **I** = *H. uvarum* 5380), *Oenococcus* spp., specific primers (**J** = *O. oeni* 4042) and *S. cerevisiae* primers (**K** = *S. cerevisiae* CECT 2056)

The detection limit is defined as the lowest concentration of cells that demonstrated amplification in gel electrophoresis and produced a visible band on the test strip. We assessed the detection limit of LAMP-LFD by conducting LAMP reactions with different serial dilutions using each set of designed primers. Positive amplification was observed in 3/3 reactions at 10⁸ CFU/mL, 3/3 at 10⁶ CFU/mL, 3/3 at 10⁴ CFU/mL, and 3/3 at 10² CFU/mL as shown in Fig. 6, and further confirmed through gel electrophoresis, as shown in Fig. 7. Based on replicate consistency, the detection limit was defined as the lowest cell-equivalent concentration that yielded positive amplification in all replicate reactions, which was determined to be 10^2^ CFU/mL. To further investigate, we tested different lower concentrations of viable cells such as 10^1^ and 10^0^ CFU/mL, in LAMP-LFD, but we didn’t get any band on strips and gel electrophoresis the results are not included in this study.

## Discussion

The increasing incidence of wine spoilage has highlighted the need for rapid, sensitive, reliable method to ensure wine quality, and stability (Bartowsky et al. 2003). Traditional approaches, including culture based microbiological techniques and PCR-based molecular assays, provide valuable information but are often limited by long processing times, high operational costs, and the presence of inhibitory compounds inherent to wine matrices (Fugelsang and Edwards 2007; Soares-Santos et al. 2017). These limitations restrict their routine application in wineries, where fast decision-making and minimal equipment requirements are desirable. Although conventional LAMP has emerged as an attractive isothermal amplification method, visualization steps involving gel electrophoresis or fluorescent dyes may still require additional equipment and can compromise ease of interpretation and detection limits.

In this study, we developed and evaluated a loop-mediated isothermal amplification coupled with lateral flow dipstick detection (LAMP-LFD) for the rapid identification of microorganisms relevant to wine fermentation and spoilage. The approach integrates broad-range Panbacteria and Panfungal primers for general microbiota surveillance with specific primers targeting *Oenococcus* spp., and *Saccharomyces cerevisiae*, two organisms of key relevance to wine stability and fermentation management. The simplified DNA preparation procedure, involving cell washing and disruption with glass beads Soares-Santos et al. (2018), allows direct sample processing without complex extraction steps, making the assay particularly suitable for on-site applications.

To further improve assay performance, gold nanoparticles (AuNPs) were incorporated in the LAMP–LFD system. This addition increased detection specificity and minimized false-positive outcomes, consistent with previous reports on nanoparticle-enhanced molecular diagnostics (Ye et al. 2018). The assay successfully detected a range of wine-relevant bacteria (*A. aceti*,* G. oxydans*, *L. plantarum* and *O. oxydans*) and yeast (*B. bruxellensis*, *H. uvarum*, *L. thermotolerans*, *M. pulcherrima* and *S. cerevisiae*).

In this study all the primers were tested with non-specific species, but they didn’t show any amplification which prove the specificity of the design primers and LAMP-LFD assay validation. The LAMP-LFD assay achieved a detection limit of 10² CFU/mL, comparable to or exceeding detection limit reported for other molecular tools applied in wine microbiology (Wachiralurpan et al. 2017; Luo et al. 2022). Compared with culture-based and microscopic approaches, which require extended incubation and skilled interpretation Fugelsang and Edwards (2007), and PCR-based methods requiring thermal cyclers and specialized laboratories Savazzini and Martinelli (2006), the developed method offers a rapid, low-cost, and equipment-minimal alternative. Importantly, the flexibility to monitor both general microbial load and specific spoilage organisms within the same methodological platform provides a practical advantage rarely achieved by previously reported assays. In addition to all these advantages, the proposed assay offers practical advantages for routine application. It requires only basic LAMP instrumentation and lateral flow strips for endpoint visualization, making it suitable for laboratories with limited resources. The complete workflow can be completed within approximately 1 h, and the per-sample cost remains low, largely depending on reagent use and LFD strip consumption.

Nevertheless, this study has certain limitations such as this study can’t quantify the wine related microorganisms and does not allow multiplexing because all amplicons are labeled with biotin/fluorescein and the LFD detects only one product at a time, but this situation is similar to PCR reactions, and multiplexing is not always necessary. Moreover, wine matrix composition (e.g., ethanol, pH, SO₂, and phenolic content) may affect DNA extraction and amplification. Although red wine was the primary matrix in this study, preliminary testing with white wine and grape must showed consistent performance with minor signal variation. Finally, LFD-based validation was conducted using *O. oeni* only, although preliminary primer validation included additional *Oenococcus* species.

Future work should focus on validation across a wider range of wine matrices and assessment across a wider range of closely related and winery-associated microbial species. In addition, developing multiplexing and LAMP-LFD quantification method for quantification of wine microorganisms. With these refinements, the LAMP-LFD platform has strong potential to serve as an accessible, reliable diagnostic tool for routine monitoring and microbial quality management in modern winemaking.

## Conclusions

This study presents a LAMP-LFD assay, enhanced with gold nanoparticles, as a rapid, sensitive, and specific tool for detecting wine spoilage microorganisms. By combining broad-range and targeted primer sets, the method enables both comprehensive microbial surveillance and precise detection of critical taxa. The assay achieved a low detection threshold, high specificity, and clear visual readout, underscoring its potential for practical use in the wine industry. Beyond its technical performance, this method addresses a critical challenge in winemaking: the timely and cost-effective detection of spoilage microorganisms. Its adoption could help reduce economic losses, maintain wine quality, and strengthen consumer confidence.

## Supplementary Information

Below is the link to the electronic supplementary material.

Supplementary Material 1

## Data Availability

Data will be provided upon request.
