# Supplementary material for: Loop-mediated isothermal amplification-lateral flow dipstick (LAMP-LFD) for detection of wine microorganisms
Source: World J Microbiol Biotechnol. 2026 Jun 5;42(6):329. doi: 10.1007/s11274-026-05066-x (PMC13236741; doi:10.1007/s11274-026-05066-x)
Supplement: Supplementary file 1 — Supplementary Material 1 [file 11274_2026_5066_MOESM1_ESM.docx]

**Supplementary Fig.**
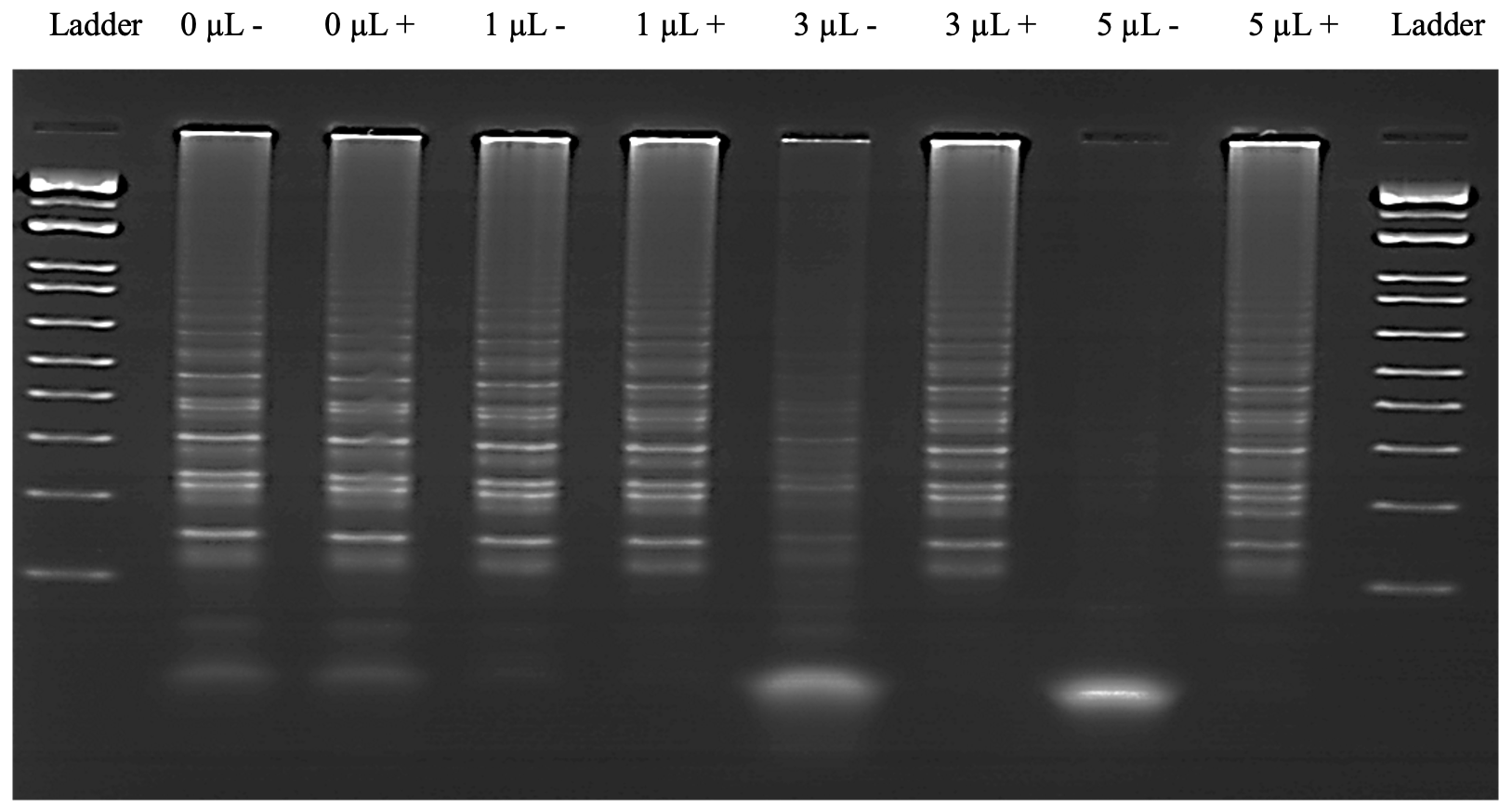
**S1** 2% agarose gel electrophoresis showing the effect of AuNPs on the LAMP reaction using Panbacteria primer with *Oenococcus oeni* - = negative control (Without DNA) + = positive (With DNA).
